# Particle beam radiation therapy using carbon ions and protons for oligometastatic lung tumors

**DOI:** 10.1186/1748-717X-9-183

**Published:** 2014-08-16

**Authors:** Nor Shazrina Sulaiman, Osamu Fujii, Yusuke Demizu, Kazuki Terashima, Yasue Niwa, Takashi Akagi, Takashi Daimon, Masao Murakami, Ryohei Sasaki, Nobukazu Fuwa

**Affiliations:** Division of Radiation Oncology, Kobe University Graduate School of Medicine, 7-5-2, Kusunoki-cho, Chuo-ku, Kobe, Hyogo, 650-0017 Japan; Department of Radiology, Hyogo Ion Beam Medical Center, Tatsuno, Japan; Department of Radiology, Hyogo College of Medicine, Nishinomiya, Japan; Department of Radiation Physics, Hyogo Ion Beam Medical Center, Tatsuno, Japan; Division of Biostatistics, Hyogo College of Medicine, Nishinomiya, Japan; Center for Radiation Oncology, Dokkyo Medical University, Tochigi, Japan

**Keywords:** Oligometastases, Lung metastases, Particle therapy, Carbon ion therapy, Proton therapy

## Abstract

**Background:**

A study was undertaken to analyze the efficacy and feasibility of particle beam radiation therapy (PBRT) using carbon ions and protons for the treatment of patients with oligometastatic lung tumors.

**Methods:**

A total of 47 patients with 59 lesions who underwent PBRT for oligometastatic lung tumors between 2003 and 2011 were included in this study. Patient median age was 66 (range, 39–84) years. The primary tumor site was the colorectum in 11 patients (23.4%), lung in 10 patients (21.3%) and a variety of other sites in 26 patients (55.3%). Thirty-one patients (66%) received chemotherapy prior to PBRT. Thirty-three lesions were treated with 320-MeV carbon ions and 26 were treated with 150- or 210-Mev protons in 1–4 portals. A median total dose of 60 (range, 52.8–70.2) GyE was delivered at the isocenter in 8 (range, 4–26) fractions.

**Results:**

The median follow-up time was 17 months. The local control, overall survival and progression-free survival rates at 2 years were 79%, 54 and 27% respectively. PBRT-related toxicities were observed; six patients (13%) had grade 2 toxicity (including grade 2 radiation pneumonitis in 2) and six patients (13%) had grade 3 toxicity. Univariate analysis indicated that patients treated with a biologically equivalent dose of 10 (BED_10_) <110 GyE_10_, had a significantly higher local recurrence rate. Local control rates were relatively lower in the subsets of patients with the colorectum as the primary tumor site. No local progression was observed in metastases from colorectal cancer irradiated with a BED_10_ ≥ 110 GyE_10_. There was no difference in treatment results between proton and carbon ion therapy.

**Conclusions:**

PRBT is well tolerated and effective in the treatment of oligometastatic lung tumors. To further improve local control, high-dose PBRT with a BED_10_ ≥ 110 GyE_10_ may be promising. Further investigation of PBRT for lung oligometastases is warranted.

## Background

In 1995, Hellman and Weichselbaum proposed a clinically significant state of metastases termed ‘oligometastases’ in which the number and site of metastatic tumors are limited [1]; this term is most often used to describe 5 or fewer metastatic lesions [2]. Patients with oligometastases have been considered candidates for curative localized therapies, such as resection or radiation therapy. Local control of an oligometastatic lesion may slow or prevent further metastatic progression, and consequently long-term patient survival can be expected [3, 4]. Surgical resection is considered as the standard option for patients with oligometastatic lung tumors, with good results in terms of local control and survival [5]. Recently, stereotactic body radiation therapy (SBRT) has been reported as an effective option for curative treatment of oligometastatic lung tumors [6–8].

Particle beam radiation therapy (PBRT), such as those involving proton and carbon ion beams, can theoretically produce a superior dose distribution in the target; this is achieved using the sharp falloff in the Bragg peaks produced by these modalities, which does not occur using photon irradiation [9]. Therefore, higher doses can be delivered without increasing toxicity to the surrounding noncancerous tissues and organs. In addition, carbon ion beams have higher relative biological effectiveness (RBE), so a higher antitumor effect can be expected [10]. Because peripheral lung tissue obeys the parallel architecture model of radiobiology [11], high-dose radiation can be focally administered without excessive risk of radiation pneumonitis, provided sufficient normal lung tissue can be spared.

PBRT results for primary lung tumors have been reported in several case series, all of which have reported good overall survival and encouraging local control rates [12–14]. However, the efficacy and feasibility of PBRT for metastatic lung lesions is unknown. In the present study, we analyzed clinical outcomes to evaluate the feasibility, efficacy and toxicity of PBRT for oligometastatic lung tumors.

## Methods

### Patient and tumor characteristics

The patient eligibility criteria for particle therapy for oligometastatic lung tumors included in this study were as follows: (1) the presence of five or fewer clinically detectable intra- and extra-thoracic metastases; (2) a locally controlled primary tumor and metastatic disease that was absent or controlled (as indicated by computed tomography (CT) and positron emission tomography (PET)-CT scans performed prior to particle therapy); (3) adequate pulmonary function; and (4) no prior radiation therapy for the target lesion. Between August 2003 and March 2011, 49 patients with 62 metastatic lung lesions were treated with PBRT using carbon ions and protons Hyogo Ion Beam Medical Center. Among them, 47 patients with 59 oligometastatic lung lesions who met the above criteria were assessable for inclusion in the study. Two patients who were excluded had progressive extra-thoracic metastatic lesions and had been treated in a palliative setting. Written informed consent was obtained from all patients, and the study was approved by the Ethics Committee at our institute. The study was conducted in compliance with the Declaration of Helsinki. Lung metastases were diagnosed based on clinical findings and diagnostic imaging including enlargement in size of the metastatic lesions and SUV enhancement regarding PET-CT scans in the majority of patients. Repeat CT and PET-CT scans were performed during regular follow-up of primary cancer disease. Thirty-six out of 37 cases with PET-CT findings showed SUV enhancement of the metastatic lesions. Enlargement in size of metastatic lesions at two or more repeated CT or PET-CT scans conducted on average at 3-month intervals were confirmed in all cases. Diagnosis of the primary tumor and metastatic lesions were confirmed by two or more diagnostic radiologists.

Patient characteristics are summarized in Table 1. Patient median age was 66 (range, 39–84) years. Twenty-three patients had one clinically detected metastatic lesion, seven patients had two, 14 patients had three, two patients had two and one patient had five located at an intra- or extra-thoracic site. The main primary tumors were colorectal cancer in 11 patients (23.4%) and non-small cell lung cancer in 10 patients (21.3%). Thirty-five patients were treated with particle therapy for single pulmonary metastases and 12 patients were treated for two pulmonary metastases. Thirty-one patients (66%) received chemotherapy prior to particle therapy. No patient received chemotherapy during the PBRT treatment period.

**Table 1:** **Patient and tumor characteristics**

Characteristics	n
Number of patients	47
Gender	
Male	29 (61.7%)
Female	18 (38.3%)
Performance status	
0	30 (63.8%)
1	13 (27.7%)
2-3	4 (8.5%)
Primary tumor	
CRC	11 (23.4%)
NSCLC	10 (21.3%)
Kidney	7 (14.9%)
Head and neck	4 (8.5%)
Others	15 (31.9%)
Prior chemotherapy	
Yes	31 (66%)
No	16 (34%)
Presence of extrathoracic disease	
Yes	24 (51.1%)
No	23 (48.9%)
Number of target thoracic metastases	
1	35 (74.4%)
2	12 (25.6%)
Tumor diameter	
<20 mm	21 (44.7%)
≥20 mm	26 (55.3%)
Reason for nonsurgical treatment	
Refusal of surgery	29 (61.7%)
Medical inoperability	
Pulmonary	10 (21.3%)
Age	7 (14.9%)
Performance status	1 (2.1%)

NSCLC, non-small cell lung cancer; CRC, colorectal cancer.

### Treatment technique

The patients were treated using 320-MeV carbon ion beams and 150-MeV or 210-Mev proton beams (Mitsubishi Electric Corporation, Tokyo, Japan). A respiratory gating irradiation system developed at the National Institute of Radiological Sciences in Chiba was used until April 2007, and an AZ-733 (Anzai Medical, Tokyo, Japan) was used from May 2007 for beam irradiation during the exhalation phase. The biological effects of both proton and carbon ion therapy were evaluated *in vitro* and *in vivo*. The RBE values for proton and carbon ions were determined to be 1.1 and 2.0–3.7 (depending on the depth of the spread-out Bragg peaks), respectively. Three-dimensional radiation therapy planning was performed using a treatment-planning machine (FOCUS-M:CMS, St. Louis, MO, USA and Mitsubishi Electric Corporation, Tokyo, Japan] until April 2008 and XiO-M [CMS and Mitsubishi Electric Corporation] from May 2008). Each patient was immobilized using a custom-made thermoplastic cast, and 2-mm-thick CT images were obtained during the exhalation phase using the respiratory gating system. The lesions under the lung window were taken as the gross tumor volume (GTV). The clinical target volume (CTV) was defined as the GTV plus a 5 mm basic margin in all directions. The planning target volume (PTV) was defined as the CTV plus a setup margin of 5 mm and an internal margin (IM) of 1–4 mm. The IM was determined according to the stability of respiration under the respiratory gating system.

Eight protocols (52.8–70.2 GyE delivered in 4–26 fractions [five fractions per week]) were used in the current study (Table 2). The protocols used for carbon ion therapy and proton therapy were established on the basis of earlier experiences with these therapies. The protocols were evaluated by the institutional review committee and subjected to modifications whenever necessary. Briefly, during the study period, the prescribed dose was escalated from 56 GyE delivered in eight fractions (95.2 GyE_10_) to 64 GyE delivered in eight fractions (115 GyE_10_). In some cases when indicated, a protocol involving 52.8 GyE delivered in four fractions was initiated to shorten the overall treatment time; however, this protocol was terminated after taking into consideration the late toxicities associated with the use of hypofractionated radiation therapy. Thirteen patients were treated with 64–70.2 GyE in 10–26 fractions, taking into consideration the proximity of organs at risk. Dose fractionation for each patient was selected after discussion involving several radiation oncologists. All radiation doses were delivered to the center of the tumor. The policy for selecting beam type was based partly on the availability of the particle beams (between April 2003 and March 2005 only proton therapy was available). In April 2005, carbon ion therapy became available, and thereafter, treatment plans for both proton therapy and carbon ion therapy were produced for every patient. Then, the dose-volume histograms were compared and the most suitable beam type was selected. In general, 1–4 portals were used for both the carbon ion and proton treatment plans.

**Table 2:** **Treatment characteristics**

Characteristics	BED_10_(GyE)	Carbon ion beam treated lesions	Proton beam treated lesions	Total
Number of lesions		33	26	59
Dose protocols				
64 GyE/8Fr	115	11	5	16
52.8 GyE/4Fr	122	9	5	14
56 GyE/8Fr	95.2	4	8	12
66 GyE/10Fr	110	4	1	5
60 GyE/8Fr	105	0	4	4
64 GyE/16Fr	89.6	2	1	3
65 GyE/26Fr	81.3	3	0	3
70.2 GyE/26Fr	89.2	0	2	2

The treatment doses used with carbon ions and protons were compared on the basis of a biologically effective dose at α/β =10 GyE (BED_10_). The BED_10_ can be obtained using the linear-quadratic (LQ) model as follows:


### Follow-up and evaluation

Follow-up imaging and toxicity evaluations were obtained at 3-month intervals. All patients were followed with either chest CT or PET-CT imaging. The local response was assessed using the Response Evaluation Criteria in Solid Tumors (RECIST). Local control was defined as the absence of local tumor failure. Local tumor failure was defined as a 20% increase in the longest diameter of the tumor within the PTV margin at consecutive CT scans. This included marginal failures occurring within 1 cm of the PTV (1.5–2.0 cm from the GTV). In some cases it was difficult to distinguish between tumor regrowth and radiation induced injury; such cases were categorized for several months as stable disease until clearly apparent tumor growth had been detected by clinical observation.

Overall survival (OS), progression-free survival (PFS) and treatment-related toxicity were also evaluated. Patients were considered to have achieved PFS if the following factors were absent: progression of an existing target or metastatic lesion; appearance of a new lesion within the lung and pulmonary lymph nodes; and distant extra-thoracic relapse or death due to cancer, whichever occurred first. All time endpoints were calculated from the initiation of PBRT. Adverse events were classified according to the Common Terminology Criteria for Adverse Events, Version 3.0.

### Statistical analysis

Statistical calculations were performed using IBM SPSS Statistics 21 software (IBM, Armonk, NY, USA). The incidence rates for local control and OS were determined using the Kaplan-Meier method. The log-rank test and the Cox proportional hazards regression model were used for univariate and multivariate analyses, respectively. Values of *P* < .05 were considered as being significant.

## Results

### Local control rates and survival analysis

**Figure 1:**
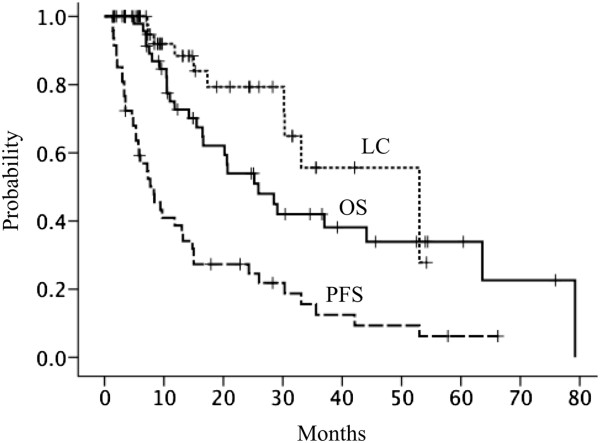
**Local control (LC), overall survival (OS) and progression-free survival (PFS) after particle beam radiation therapy for lung oligometastases.** Local control, overall survival and progression-free survival rates at 2 years were 79, 54 and 27%, respectively.

All patients were assessable for local control and survival. The median follow-up time was 17 (range, 3.5–79.2) months. Actuarial local control rates at 1 and 2 years were 88.4 and 79.3%, respectively (Figure 1). Ten events involving local failure were recorded. Eight lesions recurred within the GTV and two lesions recurred within the PTV margin. Five out of 10 local failures were metastases from colorectal cancers. The other five local failures involved renal, lung, breast, cervix and head and neck cancers.

OS rates at 1 and 2 years were 72.7 and 54%, respectively (Figure 1). PFS rates at 1 and 2 years were 38.7 and 27.3%, respectively (Figure 1). Disease progression was observed in 39 patients (83%): local failure-only occurred in five; local failure and new pulmonary recurrence in one; local failure and new extra-thoracic recurrence in four; new pulmonary recurrence-only in 11; and pulmonary lymph nodes failure-only in four. New extra-thoracic recurrence-only was observed in 14 patients: the liver in four; the bone in three; the brain in three; and the renal system in four.

### Prognostic factors (univariate and multivariate analysis)

**Figure 2:**
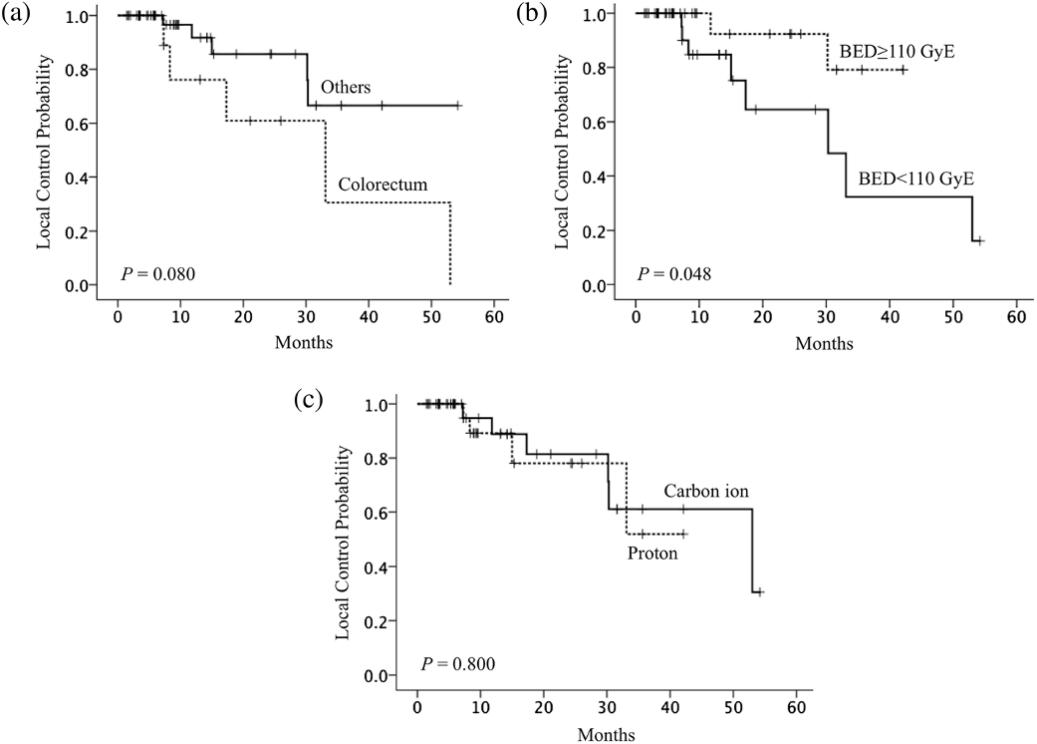
**Local control rates according to prognostic factors. (a)** primary tumor sites (colorectum, n = 11; others, n = 36); differences were marginally significant. **(b)** BED_10_ (BED_10_ < 110 GyE, n = 25; BED_10_ ≥ 110 GyE, n = 22); differences were significant. **(c)** beam type; there was no significant difference in local control rates between carbon ion therapy and proton therapy.

Univariate analysis using the log-rank test indicated that the BED_10_ (<110 GyE_10_) (*P* = .048) was a significant covariate for local failure (Figure 2 and Table 3). The correlation between the colorectum as the primary tumor site and local control rates was marginally significant (*P* = .080). All five local failures involving metastases from colorectal cancers were prescribed with dose fractionation to deliver a BED_10_ dose of <110 GyE. There was no significant difference in local control rates between carbon ion therapy and proton therapy (*P* = .800). Univariate analysis did not show a significant correlation between selected variables and survival rates. Multivariate analysis did not reveal any significant covariates for local failure (Table 3).

**Table 3:** **Assessment of prognostic factors affecting local control rates using univariate and multivariate analysis**

Variable	No. of patients	***P***value	Adjusted HR (95% CI)
*Univariate analysis*			
Age			
<60 years	17	0.956	
≥60 years	30		
Gender			
Male	29	0.396	
Female	18		
Performance status			
0	30	0.447	
≥1	17		
Primary tumor site			
Colorectum	11	0.080	
Others	36		
Tumor diameter			
<20 mm	21	0.237	
≥20 mm	26		
BED_10_			
<110 GyE	25	0.048	
≥110 GyE	22		
Beam type			
Proton	18		
Carbon ion	23	0.800	
Proton and carbon ion	6		
*Multivariate analysis*			
Primary tumor site		0.287	2.666 (0.439-16.195)
BED_10_		0.392	2.558 (0.298-21.996)

### Toxicities

Table 4 summarizes adverse events according to grade. Grade 2 or higher toxicities were observed in five patients who underwent carbon ion therapy and seven patients who underwent proton therapy. Symptomatic grade 2 radiation pneumonitis was noted in two patients (4.2%), and symptomatic grade 2 dermatitis was observed in three (6.4%). One patient developed a grade 2 rib fracture and grade 2 fibrosis of the soft tissue of the thoracic wall (2.1%). Grade 3 rib fracture was observed in one patient (2.1%). Grade 3 dermatitis was observed in five patients (10.6%). Most adverse events remained at grade 1 and there were no cases of grade 4–5 toxicity. No adverse effects regarding the spinal cord, great vessels or esophagus were observed.

**Table 4:** **Particle beam radiation therapy related toxicity**

	Grade			
Toxicity	1	2	3	4
Acute events (within 3 months post radiotherapy)				
Dermatitis	20	1	2	0
Pneumonitis	3	0	0	0
Late events (after 3 months post radiotherapy)				
Dermatitis	8	2	3	0
Rib fracture	0	1	1	0
Soft tissue damage	0	1	0	0
Pneumonitis	11	2	0	0

## Discussion

The present single-institutional clinical study investigated the role of PBRT in patients with one or two lung metastases. We believe that this is the first dedicated series involving lung oligometastases in which the therapeutic efficacy of both carbon ion and proton therapy has been reported on.

Local control of oligometastatic lesions may slow or prevent further metastatic progression; thus, long-term survival can be expected. A landmark study of >5000 patients by the International Registry of Lung Metastases demonstrated that long-term survival could be achieved in patients with lung metastases treated with metastasectomy [5]. Nevertheless, certain patients will be considered as being medically or functionally inoperable. Recently, interest has grown in SBRT as a credible alternative therapy for oligometastases, with local control rates that vary from 25 to 96% at 2 years [6, 7, 15–20]. Our institution has reported a local control rate of 79% at 2 years. Although it is difficult to make a precise comparison with published SBRT studies because of the different modalities and fractionation schedules used, it is possible to speculate on a few prognostic factors that might affect the outcome of local control.

The primary site of metastatic lung tumors may influence their local control using PBRT. Milano et al. reported the results for 293 oligometastatic lesions treated using SBRT with the preferred regimen of 50 Gy in 10 fractions (BED_10_ = 75 Gy_10_) [17]. The 2-year local control rate for all lesions was 70%, and metastatic tumors with colorectal, pancreatic and hepatobiliary origins showed significantly poorer local control rates. Takeda et al. analyzed the local control of oligometastatic lung tumors from colorectal cancer and other primary cancers relative to primary lung cancer after SBRT. The local control rate for colorectal oligometastatic lung tumors has been reported to be significantly lower than that for tumors with other origins (72 vs 94% at 2 years) [18]. In the present study, lung metastases from colorectal cancer had a poorer local control rate than metastases from other primary tumors. The reasons for these poor outcomes are unclear. We speculate that colorectal cancer metastases contain a larger proportion of necrotic and hypoxic cells as compared with other tumor types [21], and hypoxia can lead to the development of a higher grade of malignancy.

The optimal dose regimen for PBRT regarding lung metastases has not yet been determined. Several studies have analyzed dose fractionation and BED_10_ rates in SBRT for primary and metastatic lung cancers. Norihisa et al. reported that feasible dose escalation involving SBRT, from 48 Gy in four fractions (105.6 Gy_10_) to 60 Gy in five fractions (132 Gy_10_), led to improved local tumor control [7]. Hamamoto et al. performed SBRT using 48 Gy in four fractions (105.6 Gy_10_) for 12 metastatic lung tumors and reported a poor local control rate of 25% at 2 years, although they reported that the most common primary site in their study was colorectal cancer [16]. In all these reported series, fractionation doses involving a higher BED_10_ likely contributed to the higher rate of local control observed. In the present study, a higher local control rate was achieved when dose fractionation delivering a BED_10_ of <100 GyE_10_ was escalated to a BED_10_ of ≥110 GyE_10_, irrespective of the primary tumor sites. None of the lesions prescribed with a BED_10_ of ≥110 GyE_10_ showed local recurrence. As discussed previously, we speculate that colorectum as a primary site may be a risk factor for local failure, particularly when the BED_10_ for PBRT is comparatively low. Based on these results, we are currently delivering 64 GyE in eight fractions (115 GyE_10_) as our standard protocol.

In our study, pulmonary toxicity was modest using both carbon ion therapy and proton therapy. Previous SBRT studies have suggested that the lung V5 (volume of lung receiving at least 5 Gy) is the most significant factor associated with radiation pneumonitis [22]. Regarding dose-volume comparison, proton therapy has been reported to deliver a lower mean dose to the lung and lower V5 values relative to SBRT [23, 24]. The advantages offered by PBRT include providing a precise dose localization and favorable dose-depth distribution using a small number of portals; these make it useful in limiting the area of normal tissue exposed to the low-dose region. This is especially useful for patients with limited residual lung function, large lesions and centrally located lesions, particularly with regard to lung metastases where intrapulmonary recurrence is commonly detected and reirradiation may be indicated [13, 25]. The occurrence of skin toxicity in our study could be related to the use of one or two portals during the earlier period of our study. This had obtained adequate spread-out Bragg peaks but led to relatively high skin doses, particularly in lesions proximal to the chest wall. Previous photon SBRT and PBRT studies also reported the use of 2 or 3 beams as a factor associated with higher rates of skin toxicity [13, 26, 27]. We are now taking skin into consideration as an organ at risk in each radiation planning session and increase beam numbers by using up to 4 portals in order to minimize irradiation to the skin.

The uncertainty in dose conformity in PBRT as compared with SBRT has also been widely discussed [28–30]. Oshiro et al. reported that the dosimetry involving the proton beam is very sensitive to the tissue density in the beam pathway. The beam range and target coverage, especially in the case of the PTV, may be changed if the pathway is misaligned due to respiratory motion, variation in setup and tumor shrinkage [30]. Marginally worse coverage of the PTV in proton relative to photon therapy has been reported, and these uncertainties have resulted in a requirement for larger treatment planning margins in relation to proton therapy [28, 29]. However, the impact of dosimetric differences described above on clinical outcomes was not reported. In the present study, two out of 10 lesions recurred within the PTV margin. Further evaluation of clinical outcomes and toxicities to determine the need for larger treatment planning margins is now being taken into consideration.

The question remains as to whether or not aggressive radiation treatments involving patients with oligometastases have significant clinical benefits. In a systematic review regarding SBRT for 334 patients with 564 pulmonary oligometastases it was reported that high local control rates (77.9% for SBRT and 78.6% for stereotactic radiosurgery [SRS]) could potentially lead to a survival benefit [31]. Similar to our findings, the most promising results presented in this review seem to be from the use of a prescribed BED of >100 Gy at the isocenter and a BED approximating 100 Gy at the tumor periphery. However, the 2-year weighted overall survival rate was comparatively low (53.7% for SBRT and 50.3% for SRS). Because of the lack of randomized trials, the effect of stereotactic ablative radiotherapy (SABR) on survival remains unclear [31]. Moreover, although SABR is generally safe, there remains a risk of toxicity, and also a small risk of treatment related mortality [32]. A multicenter randomized phase II trial is currently being conducted to assess the impact of a comprehensive oligometastatic SABR treatment program on overall patient survival and quality of life [33]. In the future, a phase III trial will be required to demonstrate the feasibility of SABR for the treatment of oligometastatic lung tumors.

The limitations of the present study include: 1) the results were obtained retrospectively and not through randomized trials; 2) during the study period we used different treatment protocols for proton and carbon ion therapy; and 3) there was a small number of patients enrolled, and consequently the statistical power was low.

## Conclusions

Carbon ion therapy and proton therapy for oligometastatic lung tumors were effective with tolerable side effects. Higher rates of overall survival and local control, including metastases from colorectal cancers, may be achievable using high-dose PBRT with a BED_10_ ≥ 110 GyE_10_. PBRT has the advantage of sparing the contralateral and normal lung tissues. Thus, this modality could prove to be a feasible alternative for patients with impaired lung functions that are not amenable to surgery. Although further prospective randomized investigation is required, our data could serve as a basis for future refinement of an optimal PBRT regimen for lung metastases.

## Abbreviations

PBRT: Particle beam radiation therapy
BED: Biologically equivalent dose
SBRT: Stereotactic body radiation therapy
RBE: Relative biological effectiveness
PET: Positron emission tomography
CT: Computed tomography
GTV: Gross tumor volume
CTV: Clinical target volume
PTV: Planning target volume
IM: Internal margin
LQ: Linear-quadratic
RECIST: Response evaluation criteria in solid tumors
OS: Overall survival
PFS: Progression-free survival
SRS: Stereotactic radiosurgery
SABR: Stereotactic ablative radiotherapy.
